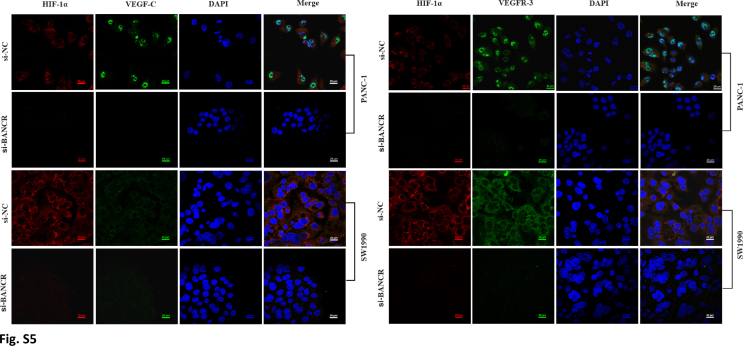# Long non-coding RNA BANCR promotes pancreatic cancer lymphangiogenesis and lymphatic metastasis by regulating the HIF-1α/VEGF-C/VEGFR-3 pathway via miR-143-5p

**DOI:** 10.1016/j.gendis.2023.05.014

**Published:** 2023-07-04

**Authors:** Shaolong Hao, Yu Ji, Weixuan Pan, Hao Sun, Fang Nie, Jonathan Ray Warren, Yuchuan Ding, Wei Han

**Affiliations:** a Department of General Surgery, Beijing Luhe Hospital, Capital Medical University, Tongzhou, Beijing 101149, China; b Department of Central Laboratory, Beijing Luhe Hospital, Capital Medical University, Tongzhou, Beijing 101149, China; c Department of Neurosurgery, Wayne State University School of Medicine, John D. Dingell VA Medical Center, Detroit, MI 48201, USA

Pancreatic cancer is one of the most aggressive malignant tumors of the digestive system. Lymph node metastasis (LNM), as the main way of invasion and metastasis, can occur in the early stage of pancreatic cancer and be considered the most important factor affecting the clinical treatment and prognosis of patients with pancreatic cancer. Previous studies have found that the incidence of lymphatic invasion of cancer cells is 3–5 times higher than that of vascular invasion.^1^ Although LNM of pancreatic cancer has obvious clinical importance, the specific molecular mechanism is still unclear.

Lymphangiogenesis has been proven to be the key process in the LNM of malignant tumors. Previous studies have found that the VEGF-C/VEGFR-3 axis plays a central role in initiating lymphangiogenesis.^2^ In this study, we compared the expression of VEGF-C and VEGFR-3 in tumor tissues of pancreatic cancer patients who with and without LNM. The results showed that there was a co-localized expression of VEGF-C and VEGFR-3 in tumor tissues of pancreatic cancer patients with LNM, and the level of expression was significantly higher than that in patients without LNM (Fig. S1). Other studies portend similar findings. Ochi et al found that there is a significant correlation between VEGF-C and VEGFR-3 expression in tumor tissues of patients with pancreatic cancer. Their data suggest that the combination of VEGFR-3 and VEGF-C can stimulate lymphangiogenesis in tumors, induce tumors to produce new lymphatic capillaries, and increase the risk of pancreatic cancer LNM.^3^ Based on our results and previous research, the VEGF-C/VEGFR-3 pathway plays a core role in lymphangiogenesis and LNM of pancreatic cancer.

Long non-coding RNA (lncRNA) is abnormally expressed in a variety of human malignant tumors and can directly or indirectly participate in the process of tumor proliferation, invasion, and metastasis. Though some signaling cascades in lymphangiogenesis have been studied, there is a dearth of data assessing the role of BANCR. In recent years, some studies have also found that BANCR is involved in the proliferation, metastasis, and invasion of human malignant tumors such as gastric cancer, liver cancer, and thyroid cancer. Additionally, a lot of studies have shown that BANCR mutations are also associated with pancreatic cancer tumorigenesis, however, the regulation of BANCR in pancreatic cancer and its mechanism are not clear. In this study, qRT-PCR was used to detect the expression of BANCR in 36 pairs of pancreatic cancer tumor tissues and adjacent normal pancreatic tissues (Fig. 1A). The results showed that the level of BANCR in pancreatic cancer was significantly increased (2.644 ± 0.24 *vs.* 1.252 ± 0.136). Then, we further detected the expression of BANCR in pancreatic cancer cell lines (SW1990 and PANC-1) and HPDC. The results showed that the expression levels of BANCR in the SW1990 (3.988 ± 0.7) and PANC-1 (5.338 ± 0.975) were significantly higher than that in HPDC (1.013 ± 0.109) (Fig. S2). Combined with the statistical analysis of the clinicopathological data of the patients (Table S1), we found that the level of BANCR was positively correlated with survival prognosis, LNM, and micro-lymphatic vessel density (MLVD) in tumor tissue. Further analysis revealed that BANCR was higher in the tumor tissues of pancreatic cancer patients with LNM (Fig. 1B). Furthermore, lymphatic vessel-specific antibody D2-40 and immunohistochemical fluorescence staining were used to analyze the correlation between MLVD and LNM in 36 patients with pancreatic cancer at the clinical tissue sample level. Data suggests that MLVD in pancreatic cancer tumor tissues with LNM is significantly higher than in tissues without LNM (Fig. 1C) and the level of BANCR is closely related to the number of MLVD (Fig. 1D) in patients with pancreatic cancer. We further analyzed the expressions of VEGF-C and VEGFR-3 in pancreatic cancer tumor tissues of the LNM group and the non-metastasis group. We found that the levels of VEGF-C and VEGFR-3 in pancreatic cancer tumor tissues of the LNM group were significantly higher than those in the non-metastasis group (Fig. S1). In the present work, BANCR was positively correlated with expression levels of VEGF-C and VEGFR-3 in pancreatic cancer tumor tissues (Fig. S3)*.* This evidence suggests that BANCR in pancreatic cancer tissues may promote lymphangiogenesis and LNM by up-regulating the expression of lymphangiogenesis factors VEGF-C and VEGFR-3.

**Figure 1:**
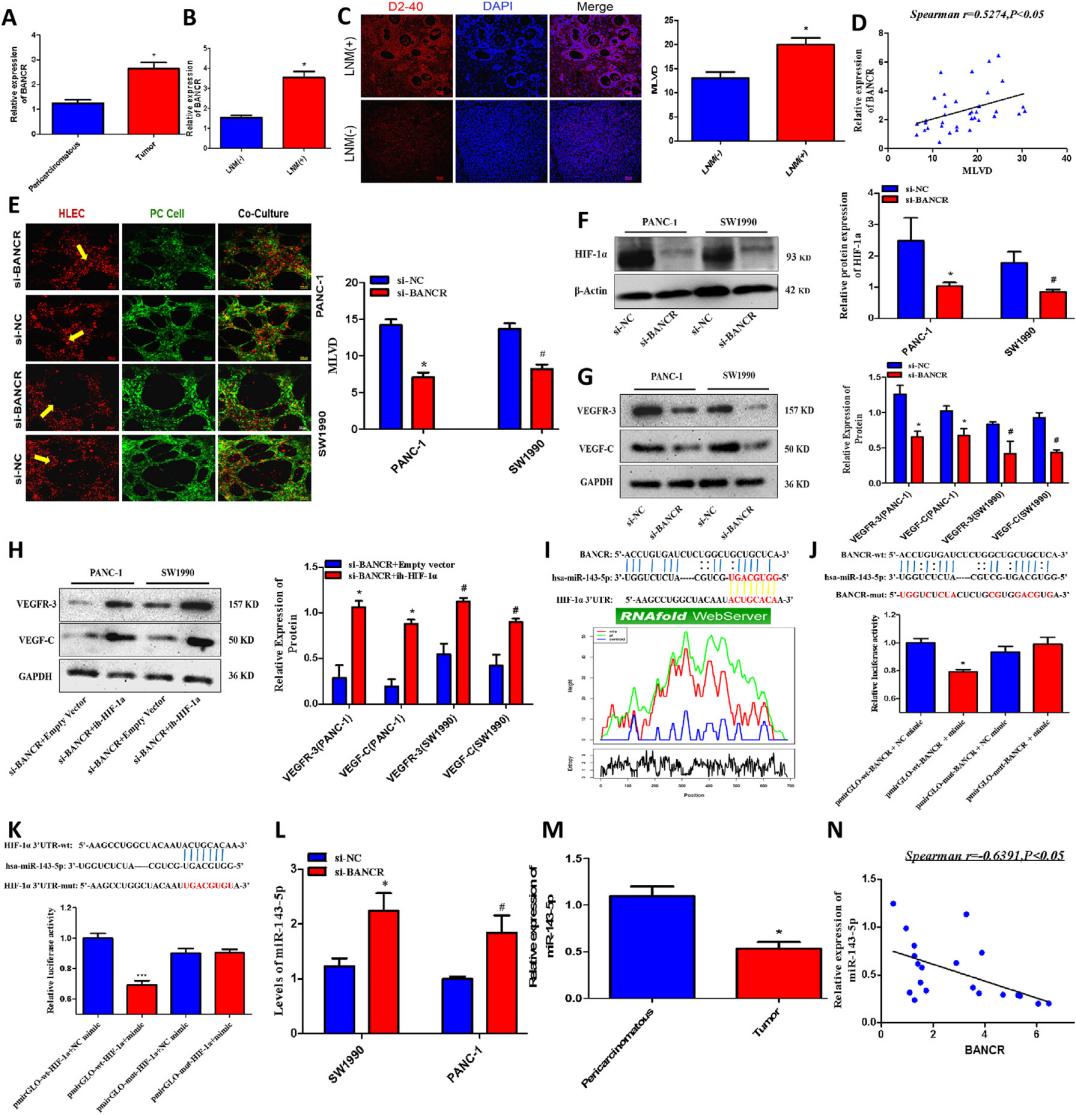
BANCR promotes pancreatic cancer (PC) lymphangiogenesis and lymphatic metastasis by up-regulating the HIF-1α/VEGF-C/VEGFR-3 pathway via sponging miR-143-5p. **(A)** Bar graph representation of the differences in BANCR levels in PC tumor tissues and peri-carcinoma pancreatic tissues. **(B)** Bar graph representation of the changes in BANCR levels in PC tumor tissues between PC patients with lymph node metastasis (LNM^+^) and without lymph node metastasis (LNM^–^). **(C)** Immunofluorescence analyses of micro-lymphatic vessels in tumor tissues of PC patients with and without LNM were performed with the D2-40 antibody. Nuclei were stained with DAPI. Bar graph representation of the differences in micro-lymphatic vessel density (MLVD) in PC tumor tissues between PC patients with and without LNM. **(D)** The correlation between MLVD and BANCR levels in tumor tissues of PC patients. **(E)** Representative images (magnification, ×20) of lymphangiogenesis experiments using 3D co-culture PC cell lines (SW1990 and PANC-1 cells) and lymphatic endothelial cells. The PC cells were labeled with green fluorescence and the lymphatic endothelial cells were labeled with red fluorescence. The micro-lymphatic vessels as red rings are marked with yellow arrows. Quantitative analysis of the changes of MLVD in si-BANCR and si-NC transfected PC cell lines. **(F)** Western blot analysis of the effect of BANCR knockdown on HIF-1α protein expression in PANC-1 and SW1990 cells. **(G)** Western blot analysis of the effect of BANCR knockdown on VEGF-C and VEGFR-3 protein expression in PANC-1 and SW1990 cells. **(H)** Western blot analysis of the effect of further overexpression HIF-1α after BANCR knockdown on VEGF-C and VEGFR-3 protein expression in PANC-1 and SW1990 cells. **(I)** Bioinformatics analysis predicted the specific binding sites of miR-143-5p to BANCR and HIF-1α. **(J)** Double luciferase reporter gene assays showed the luciferase activity of wild-type or mutant BANCR following co-transfection with either the miR-143-5p or control mimics. Relative firefly luciferase expression was normalized to that of renilla luciferase. **(K)** Double luciferase reporter gene assays showed the luciferase activity of wild-type or mutant HIF-1α mRNA following co-transfection with either the miR-143-5p or control mimics. Relative firefly luciferase expression was normalized to that of renilla luciferase. **(L)** The changes of miR-143-5p in PC cells (PANC-1 and SW1990) after BANCR knockdown. **(M)** The expression of miR-143-5p in PC tissues and peri-carcinoma pancreatic tissues. **(N)** The correlation between miR-143-5p and BANCR levels in tumor tissues of PC patients. ^∗^*P* < 0.05, ^#^*P* < 0.05.

In order to clarify the effect of BANCR on pancreatic cancer lymphangiogenesis, we performed lymphangiogenesis experiments using 3D co-culture technology of pancreatic cancer cells and lymphatic endothelial cells (Fig. 1E). Compared with SW1990 and PANC-1 cells in the empty plasmid control group (si-NC), the BANCR knockdown group (si-BANCR) showed significant reductions in lymphangiogenesis among pancreatic cancer cells (3.87 ± 0.46 *vs.* 18.00 ± 1.58, *P* < 0.05). The expression of HIF-1α/VEGF-C/VEGFR-3 pathway molecules in cells of the two groups was further measured by Wesern-blot and qRT-PCR. The results showed that the expression of HIF-1α (Fig. 1F), VEGF-C, and VEGFR-3 (Fig. 1G) in the si-BANCR group was significantly lower than those in the si-NC group (*P* < 0.05). In order to confirm the effect of BANCR on VEGF-C/VEGFR-3 pathway by regulating HIF-1α, we over-expressed HIF-1a after knocking down BANCR (Fig. S4) and observed the localization and expression of HIF-1α, VEGF-C, and VEGFR-3 in the si-BANCR group and si-NC group by immunofluorescence staining (Fig. S5). These results also showed that the levels of HIF-1α, VEGF-C, and VEGFR-3 in the cytoplasm decreased simultaneously after BANCR knockdown. In addition, the expression of VEGF-C and VEGFR-3 can be restored at the transcription and translation levels with further overexpression of HIF-1α after BANCR knockdown (Fig. 1H). Previous studies have shown that the over-expression of HIF-1α in pancreatic cancer is related to tumor progression and LNM. Tao et al also assessed the role of HIF-1α and VEGF-C in pancreatic cancer. In their cohort of 75 patients with pancreatic head cancers, they found a positive correlation of HIF-1α with the expression of VEGF-C where they portend that HIF-1α may promote lymphangiogenesis and LNM in patients with pancreatic cancer.^4^ Based on our data and the above studies, we have reason to believe that BANCR can promote pancreatic cancer lymphangiogenesis by up-regulating the HIF-1α/VEGF-C/VEGFR-3 pathway. These results suggest that BANCR can promote the levels of VEGF-C and VEGFR-3 by up-regulating HIF-1α and then promoting lymphangiogenesis and LNM in pancreatic cancer.

In order to clarify the regulatory mechanism of BANCR on HIF-1α, we found that there were specific binding sites between BANCR and miR-143-5p and that miR-143-5p could competitively bind to inhibit the expression of HIF-1α with bioinformatics analysis (Fig. 1I) and double luciferase reporter genes (Fig. 1J, K). In the present study, the negative regulation of miR-143-5p on HIF-1α was further verified and BANCR knockdown significantly up-regulated the level of miR-143-5p in PC cell lines (Fig. 1L). At the same time, we examined the expression of miR-143-5p in PC tumor (Fig. 1M) and para-tumorous tissues and analyzed the correlation between the levels of BANCR and miR-143-5p in PC tumor tissues (Fig. 1N). As expected, there was a significant negative correlation between BANCR and miR-143-5p expression in pancreatic cancer tumor tissues (Spearman *r* = −0.6391, *P* < 0.05). Similarly to our results, Wang et al assessed the role of miR-143-3p in pancreatic cancer and found that it attenuates apoptotic pathways, suppresses cell growth, and reduces the invasiveness of pancreatic cancer.^5^ These data suggest that BANCR can up-regulate the HIF-1α/VEGF-C/VEGFR-3 pathway through competitive inhibition of miR-143-5p, promote pancreatic cancer micro-lymphangiogenesis, and promote pancreatic cancer LNM which may serve as a potential diagnostic and therapeutic target for future treatment of pancreatic cancer.

## Conflict of interests

The authors have no relevant affiliations or financial involvement with any organization or entity with a financial interest in or financial conflict with the subject matter or materials discussed in the manuscript. This includes employment, consultancies, honoraria, stock ownership or options, expert testimony, grants or patents received or pending, or royalties.

## Funding

This work was supported by the Capital Health Development Scientific Research Project (China) (No. 2022-2-7081), Beijing Natural Science Foundation (China) (No. 7234377), and Science and Technology Plan Project of Tongzhou District, Beijing, China (No. KJ2022CX016).

## Figures and Tables

**figs1Immunofluorescence analyzed the localization and expression of VEGF-C and VEGFR-3 in PC patients' tumor tissues with and without lymph node metastasis (LNM). Nuclei were stained with DAPI. Bar graph representation of the differences in VEGF-C and VEGFR-3 expression in PC tumor tissues between PC patients with and without LNM. ∗P<0.05:**
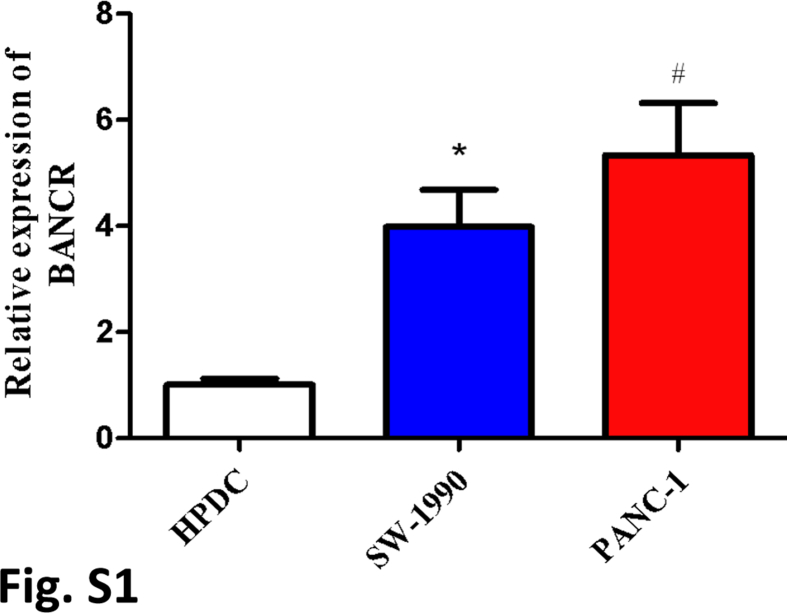


**figs2The background expression of BANCR in HPDC, SW1990, and PANC-1 cells. ∗P < 0.05, #P < 0.05:**
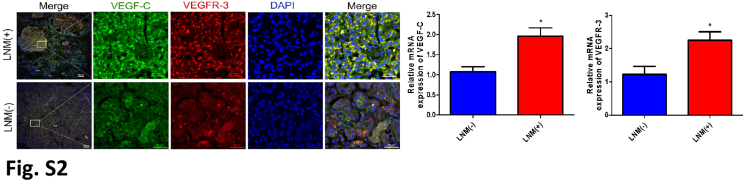


**figs3The correlation between relative mRNA expression of VEGF-C (blue line) and VEGFR-3 (red line) and BANCR levels in PC tumor tissues:**
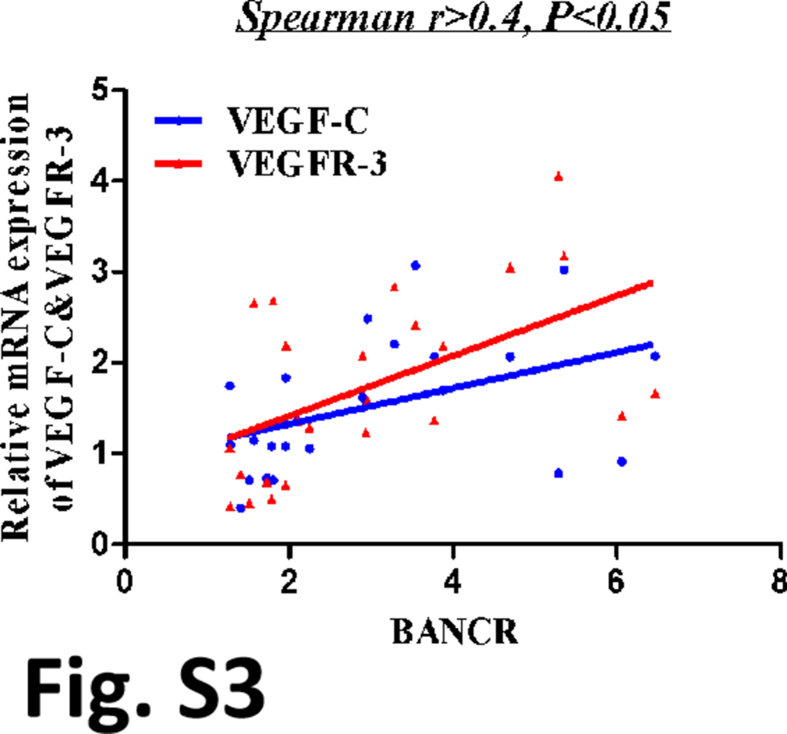


**figs4Western blot (A) and qRT-PCR (B) for verifying the successful over-expression of HIF-1α after BANCR knockdown in PANC-1 and SW1990 cells:**
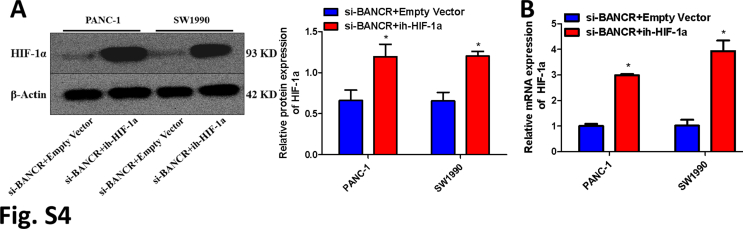


**figs5Immunofluorescence assay showed the localization and expression of HIF-1α VEGF-C and VEGFR-3 in PANC-1 and SW1990 cells after BANCR knockdown:**