# Supplementary material for: Long non-coding RNA BANCR promotes pancreatic cancer lymphangiogenesis and lymphatic metastasis by regulating the HIF-1α/VEGF-C/VEGFR-3 pathway via miR-143-5p
Source: Genes Dis. 2023 Jul 4;11(4):101015. doi: 10.1016/j.gendis.2023.05.014 (PMC10937517; doi:10.1016/j.gendis.2023.05.014)
Supplement: Multimedia component 1 [file mmc1.doc]

| **Table 1. Relationship between clinicopathological characteristics and the level of BANCR in PC tumor tissues** | | | |
| --- | --- | --- | --- |
| **Parameter** | **NO. of patients (n=36)** | **The Level of BANCR (Mean ± SD)** | **P Value** |
| **Sex** |  |  |  |
| Male | 23 | 2.766±1.410 | 0.507 |
| Female | 13 | 2.428±1.531 |  |
| **Age (years)** |  |  |  |
| ＞60 | 26 | 2.845±1.553 | 0.109 |
| ≤60 | 10 | 2.122±0.986 |  |
| **Tumor Size (cm)** |  |  |  |
| T1 | 6 | 1.971±1.011 | 0.017 ***** |
| T2 | 14 | 2.263±1.389 |  |
| T3 | 8 | 2.465±1.016 |  |
| T4 | 8 | 3.995±1.501 |  |
| **Lymph Node Metastasis** |  |  |  |
| Yeas | 20 | 3.538±1.330 | ＜0.01 ***** |
| No | 16 | 1.527±0.467 |  |
| **Distant Metastasis** |  |  |  |
| Yeas | 6 | 2.871±0.940 | 0.578 |
| No | 30 | 2.599±1.532 |  |
| **Histological Grade** |  |  |  |
| Well | 9 | 1.726±0.822 | 0.037 ***** |
| Moderate | 22 | 2.789±1.237 |  |
| Poor | 5 | 3.658±2.348 |  |
| **Tumor Stage** |  |  |  |
| I | 14 | 1.630±0.779 | ＜0.01 ***** |
| II | 11 | 3.012±1.349 |  |
| III | 5 | 4.404±1.630 |  |
| IV | 6 | 2.871±0.940 |  |
